# Affective State Recognition in Livestock—Artificial Intelligence Approaches

**DOI:** 10.3390/ani12060759

**Published:** 2022-03-17

**Authors:** Suresh Neethirajan

**Affiliations:** Farmworx, Adaptation Physiology Group, Animal Sciences Department, Wageningen University and Research, 6700 AH Wageningen, The Netherlands; suresh.neethirajan@wur.nl

**Keywords:** animal emotions, animal welfare, sensors, animal-based measures, affective states, emotion modelling

## Abstract

**Simple Summary:**

Emotions or affective states recognition in farm animals is an underexplored research domain. Despite significant advances in animal welfare research, animal affective state computing through the development and application of devices and platforms that can not only recognize but interpret and process the emotions, are in a nascent stage. The analysis and measurement of unique behavioural, physical, and biological characteristics offered by biometric sensor technologies and the affiliated complex and large data sets, opens the pathway for novel and realistic identification of individual animals amongst a herd or a flock. By capitalizing on the immense potential of biometric sensors, artificial intelligence enabled big data methods offer substantial advancement of animal welfare standards and meet the urgent needs of caretakers to respond effectively to maintain the wellbeing of their animals.

**Abstract:**

Farm animals, numbering over 70 billion worldwide, are increasingly managed in large-scale, intensive farms. With both public awareness and scientific evidence growing that farm animals experience suffering, as well as affective states such as fear, frustration and distress, there is an urgent need to develop efficient and accurate methods for monitoring their welfare. At present, there are not scientifically validated ‘benchmarks’ for quantifying transient emotional (affective) states in farm animals, and no established measures of good welfare, only indicators of poor welfare, such as injury, pain and fear. Conventional approaches to monitoring livestock welfare are time-consuming, interrupt farming processes and involve subjective judgments. Biometric sensor data enabled by artificial intelligence is an emerging smart solution to unobtrusively monitoring livestock, but its potential for quantifying affective states and ground-breaking solutions in their application are yet to be realized. This review provides innovative methods for collecting big data on farm animal emotions, which can be used to train artificial intelligence models to classify, quantify and predict affective states in individual pigs and cows. Extending this to the group level, social network analysis can be applied to model emotional dynamics and contagion among animals. Finally, ‘digital twins’ of animals capable of simulating and predicting their affective states and behaviour in real time are a near-term possibility.

## 1. Quantified Animal Welfare—A Perception or a Reality?

Public concern for animal welfare is growing [1], supported by mounting scientific evidence that many animals, including mammals and birds, are capable of experiencing affective states such as fear, frustration, and joy [2,3,4]. Farm animals constitute more than 90% of the non-human animal population, in total numbering over 70 billion, two-thirds of which are farmed intensively [5]. As the global demand for animal products increases, livestock farming is expanding in step, with ever larger farms and numbers of animals to care for. Monitoring and responding effectively to livestock disease and distress is an ever-growing challenge for farmers, one that has impacts both ethically and economically [1,6].

As an animal’s affective state responds so rapidly to its physiological state and events in the external environment, it is a highly sensitive indicator of animal wellbeing [4]. Therefore, farmers who are better informed about their animals’ affective states are empowered to respond more rapidly and effectively to preserve livestock welfare and to mitigate losses in productivity and quality caused through disease and distress. However, at present, our ability to identify, quantify, and predict affective states in animals, especially positive states [7,8], is limited [7,9,10].

Although animals cannot express emotions in the same way as humans, using language, they nevertheless communicate their affective state via alternative means, including vocalizations [8,11], body and tail movements [12,13], facial expression [14,15,16,17] (including movements of ears, lips or eyes) [9,16,18], body posture, which is somewhat different from body movements, hair and or feather movements (e.g., piloerection in some mammals and raising or lowering the feathers in the crests of some birds) [19]. In addition to these behavioural signals, animal affective states are associated with changes in physiological parameters, such as heart rate [18,19,20,21], respiratory rate [21,22], and the temperature of the whole or parts of the body [23,24,25]. Furthermore, emotional changes in animals also modulate biochemical signals, such as levels of cortisol [20,26,27], lactate [27,28], and oxytocin [29] in blood and saliva.

Traditional methods for assessing farm animal affective states, such as surveys or blood sampling, cause interruptions to farming processes, and are time-consuming, making them impractical to carry out on large numbers of animals, or they involve subjective judgements that can introduce bias [30]. Currently, however, there are insufficient scientifically validated standards available for measuring and quantifying farm animal affective states based on any of these signals [9]. Furthermore, there are no established standards of good welfare in animals, rather only those relating to indicators of poor welfare, such as injury, pain and fear [14,31]. Positive affective states in farm animals are essential to monitor and foster, not only because they indicate good physical and mental health, but also because they can increase the productivity of livestock and the quality of products obtained from them [6,32]. Therefore, developing quantitative measures of farm animal affective state [33] to improve animal welfare could benefit both livestock and farmers by mitigating disease, reducing suffering, and increasing the quality and quantity of livestock output.

An emerging disruptive approach in livestock farming, currently in development within our research group at Wageningen University, is the use of biometric sensor technology in combination with big data analytics to analyse farm animal behaviour [9]. At present, no such systems have been developed for measuring and predicting farm animal affective states. Traditional methods of farm animal monitoring are time-intensive, can only be performed sporadically, and yield small data sets, making it difficult to derive accurate inferences about animal behaviour and affective state [33]. In contrast, wearable and environmental sensors allow for huge data sets of physical, biological, and biometric parameters to be continuously and concurrently acquired from individual animals or entire herds [34]. Big data methods, such as artificial intelligence (AI) and machine learning (ML) algorithms, provide a powerful, automated approach to analysing these data in real time [33,35,36]. Big data analytics thrive on a continuous flow of data, gradually learning features and patterns in the data, and improving the ability over time to accurately classify, quantify, and predict affective states [37,38,39,40,41,42].

A wide variety of both invasive and non-invasive sensor types have been developed applicable to monitoring animal behaviour and affective states [9,43]. Invasive sensors are either implanted or swallowed by the animal, and hence have the drawbacks in terms of animal welfare and harm: risking infection, and inducing stress, and hence potentially skewing the collected data. Therefore, non-invasive sensors are generally preferred for animal welfare monitoring [9]. These include remote sensors such as visual and thermal cameras, microphones, and wearable sensors attached to the animal, such as for monitoring heart and respiratory rate, and activity levels.

Small-scale studies, including from our group, have previously explored automated affective state measurement from data collected from one or a small number of sensors; the potential of big data to characterize animal affective state across multiple visual, thermal, auditory, physiological, and biochemical modalities is as yet, untapped [9,44]. The integration/fusion of multimodal sensor data is key to measuring emotional changes with sufficient speed and accuracy to be relevant for guiding livestock welfare decisions in real-world farming situations [9]. Recent innovations in AI and ML methods have substantially improved our ability to identify and quantify affective states in biometric sensor data collected from humans and animals [37,40,41,45]. These technical achievements augur a new era in farm animal welfare monitoring, in which big data analytics methods capitalize on multimodal sensor data to substantially advance our understanding of animal affective states and wellbeing [9,34]. This approach has the potential to revolutionize livestock farming by allowing farmers to continuously monitor their animals’ welfare [9], respond quickly to prevent disease and distress [34,46], and optimize care at the level of individual animals [47,48].

This perspective and critical review article provides a framework for quantitative and objective assessment of distinct affective state features and categories in farmed animals (predominantly pigs and cows). In this article, I propose ways to identify robust predictors of farm animal affective state and behaviour by collecting multimodal biometric sensor data from real farm environments; methods to develop objective, scientifically validated scales and indices of animal welfare to predict affective state and behaviour at the individual and herd levels; and perspectives to generate digital twin (digital replica of a real-world entity) models of animals that allow measured and predicted affective states and behaviours to be reported in real time to animal caretakers in an interactive and intuitive way.

## 2. Multi-Dimensional Sensor Data for Monitoring of Affective States

The Three Circles model of animal welfare proposes that the essential criteria are the basic health and functioning, natural living, and affective states of animals [49]. Although much attention has been paid to physical indicators of health, functioning, and the living conditions of livestock, limited research to date has focused on measuring their affective states [9,30]. Research into non-human animal affective states is considerably less developed than that into human affective states [7], although overlaps exist in the conceptual frameworks used to study affective states across species, especially within mammals. According to one widely accepted contemporary definition [7], emotions are short-lived affective states involving simultaneous behavioural, physiological, cognitive and subjective processes. Emotional experiences typically are characterized by two main dimensions: valence (pleasant to unpleasant) and arousal (high to low energy). Discrete emotions, such as fear, anger, joy or contentment, can be placed within this two-dimensional “affective space” [4,50]: for example, fear is negative valence–high arousal, while contentment is positive valence–low arousal.

However, while many of the existing methods for measuring emotion-related behavioural and physiological processes in animals are sensitive to arousal, they do not accurately quantify valence [7,51]. For example, sampling of cortisol levels from blood is an excellent method for quantitatively measuring arousal but is much less informative regarding valence. Measuring valence is key to improving animal welfare, as maintaining positive affective states has been shown to increase both health and happiness in animals [31,52]. Studies suggest that animals, like humans, experience affective states at multiple timescales, ranging from transient affective states to longer-lasting moods, and even relatively stable emotional dispositions akin to personality [53,54,55,56]. However, existing methods of assessing affective state in farm animals are time-consuming and fail to capture dynamic changes in affective states over time [14,30], which are key to building a comprehensive understanding of the complex factors influencing animal welfare and developing effective interventions to mitigate disease and distress. This is especially important in stressful situations, such as during transportation and preparation for slaughter [57,58,59], in which understanding how affective states are triggered in animals by environmental stimuli such as calls from other animals are vital to maintaining their welfare.

Sensor technology advances promise to empower farmers to exploit these moment-to-moment changes in the affective states of their animals, thereby increasing their wellbeing and productivity [9]. A variety of non-invasive sensors are deployed in farm environments to monitor physical, behavioural, physiological, and biochemical cues that correlate with affective states in farm animals [9,33,34]. Research into automated affective state classification and measurement in humans has led to the development of AI and ML methods suitable for analysing data from each of the different modalities such as facial features, vocalization, gait, posture, physiological data, thermal data, activity. Below, I discuss key sensor types used in farm settings, the aspects of emotional expression they measure, and the state-of-the-art in AI and ML methods for quantifying affective states based on data from each sensor type.

### 2.1. Visual Sensors—Facial Features and Expression

Using video cameras, the entire facial expression, or the appearance of particular facial features, such as the eyes, ears, nose (snout), cheeks, or jaw, can be monitored. The movement and attitude of specific facial features have been noted to reflect affective state in farm animals, for instance, backward-pointing ears indicates fear in pigs [16]. However, at present no systematic analyses of facial expressions in farm animals have been conducted [9,15,17]; therefore, an AI/ML big data approach to affective state recognition and analysis can radically deepen our understanding of correlations between facial features and affective states in farm animals.

In addition to analysing specific facial features, facial expression in animals can be assessed quantitatively using the Facial Action Coding System (FACS) [60], in which the activities of facial muscles or groups of muscles are manually scored to identify specific affective states. FACS, which was originally designed to analyse affective states on human faces, has been adapted to develop Grimace Scales [31], which quantify pain-related facial expressions in animals undergoing unpleasant procedures, such as tail docking or castration [14,61,62,63,64].

### 2.2. Thermal Imaging Cameras—Body Temperature

Thermal (infrared) imaging cameras can measure the peripheral temperature of the whole or parts of an animal’s body [65,66,67], which correlates with changes in affective state and autonomic nervous system activity [21,24,68]. A decrease in the peripheral temperature of a particular body part followed by an increase in core temperature may indicate a change in affective state [66,67]. For example, in sheep a drop in nasal temperature indicates a change in emotional valence (from negative to positive, and vice versa) [23]. To date, studies using thermal imaging cameras have not fully exploited the potential for automated real-time processing of these data. Hence, there is a need for application of image-based ML methods, such as convolutional neural networks [69,70], to improve the sensitivity, accuracy and speed of temperature-derived measures of affective states in farm animals. Convolutional Neural Network (CNN) is a deep learning algorithm and a class of artificial neural network typically used for analysing images.

### 2.3. Microphones—Vocalisations

Microphones installed in the farm environment can capture and discriminate vocalisations from many animals simultaneously, making them a powerful tool for monitoring affective state. Vocalisations have been demonstrated to convey information about affective states in a wide range of farm animals [8,71,72], including pigs [73], cows [74] and chickens [75]. Vocalizations are often involuntary, especially those indicating negative affective states, and so are good indicators of immediate emotional reactions in animals [9]. ML approaches to farm animal sound analyses have been extensively explored and successfully applied to analysing vocalizations from pigs [76], chickens [75,77] and cows [78,79].

### 2.4. Heart Rate Monitors—Heart Rate and Heart Rate Variability

**Figure 1:**
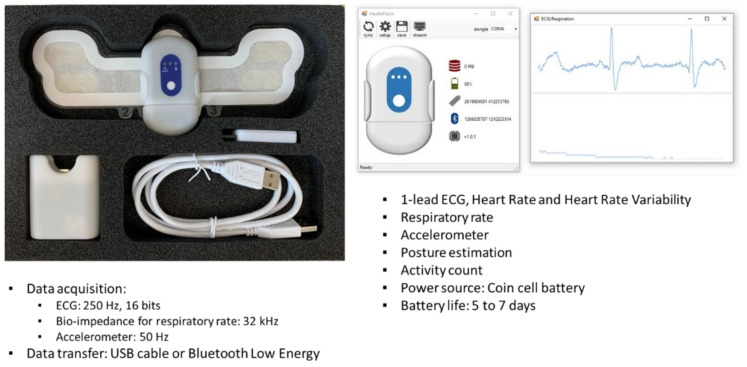
Photo of the wearable 3-in-1 sensor patch for measuring heart and respiration rates, and activity simultaneously (Source: TNO Holst Centre, The Netherlands).

Wearable heart monitoring devices for farm animals are beginning to emerge that can provide continuous monitoring of heart rhythm via electrocardiographic (electrical) or photoplethysmographic (optical) methods [80]. The heart rate and the variability in inter-beat intervals, known as heart rate variability (HRV), provide physiological measures (Figure 1) of affective state reflecting the activity of the autonomic nervous system [19,81,82]. For example, cows undergoing a stressful veterinary procedure show a decrease in HRV, accompanied by an increase in serum and saliva levels of the stress-related hormone cortisol [20]. An attempt to collect heart rate and HRV continuously from cows and pigs using the novel wearable TNO Holst Centre 3-in-1 patch, which measures heart rate, respiratory rate, and activity level simultaneously would be a break-through and will open up multimodal data collection possibilities.

### 2.5. Accelerometers—Body Movement

Accelerometers embedded in wearable devices attached to livestock allow data to be collected corresponding to an animal’s three-dimensional movement patterns [83,84,85]. Analyses of these data can yield valuable insights into the behaviour, health, and welfare of livestock [86]. ML analyses of activity data have been widely applied to successfully identify specific disease states in animals, such as lameness in sheep [87], and to accurately distinguish between multiple behaviours, such as grazing, lying down, ruminating, standing and walking in cows and sheep [88,89,90]. However, despite their extensive use to investigate and analyse farm animal behaviour, the potential contribution of activity monitors to estimating affective states in farm animals has not been developed [9,91].

### 2.6. Respirometers—Respiratory Rate

The velocity or depth of respiration is an indicator of changes in affective state [92,93,94], but this measure is confounded by many factors, including activity level, milk yield, pregnancy, ambient heat levels, and pathological conditions [95]. Therefore, respiratory rate analysis works best when combined with other physiological measures, especially heart rate [93] given the close relationship between the circulatory and respiratory systems. Traditionally, manual observations of flank movements have been used to measure respiratory rate in farm animals, but these are very time-consuming. To address this, electronic respiratory rate sensors are now being developed for a range of farm animals, including cows [22] and pigs [21], such as the sensor incorporated into the wearable TNO Holst Centre 3-in-1 patch.

The range of sensors available offer the opportunity to collect data from many different modalities simultaneously. However, analysis of data from any one sensor type alone is insufficient to accurately measure affective states in farm animals; therefore, a multimodal approach to affective state estimation is key, but this is yet to be realized in livestock farming [9]. Combining sensors across multiple modalities has the potential to significantly advance our capacity to estimate affective states and could yield valuable data and insights into affective states across species and the interrelationship between environmental factors and affective states [9,44,96]. However, to harness the tremendous potential of real-time multimodal sensor data to deepen our understanding of affective states, novel methods must be developed to collect, integrate, and analyse these data.

To address this challenge, there is an urgent and immediate need for the development and delivery of next-generation technology that integrates and analyses cues from multiple sensors simultaneously to quantify and predict affective states in farm animals. To achieve this, AI and ML methods can be applied to analyse the large data sets of high temporal resolution, multidimensional data generated by the distributed, multimodal sensor network [37,40,41,42,44,96]. This innovative application of a big data analytics approach will enable faster, more accurate and more sensitive monitoring of affective state and welfare in farm animals compared with traditional methods, meeting the challenge of identifying uncharacterized mental and affective states at high temporal resolution. By capitalizing on the immense potential of biometric sensors and computational methods, the methodologies and instrumentation aspects will substantially advance welfare standards and help caretakers to respond more effectively to maintain the welfare of their animals [34,35,43,86]. This quantitative assessment of affective states will open the door to exploring the interrelationship between environmental factors and affective states. The critical insights gained into the mechanisms underlying emotional processing should be of significance in developing tools to enhance animal welfare and in advancing our understanding of animal–human interactions.

## 3. Modelling Farm Animal Affective State and Behaviour Using Multimodal Sensor Fusion

High-fidelity, integrated multimodal imaging and sensing technologies have the potential to revolutionize how livestock are monitored and cared for [33,34]. Currently, there are no commercially available multimodal biosensing platforms capable of monitoring the affective and behavioural states of farm animals in real time [9]. Developing such a platform would allow comprehensive quantitative analyses of these states, potentially leading to significant insights and advances in our understanding of optimal approaches to animal care. The development and integration of next-generation multimodal sensor systems and advanced statistical methods to estimate and predict affective and behavioural states in farm animals would significantly open pathways for enhancing animal welfare.

**Figure 2:**
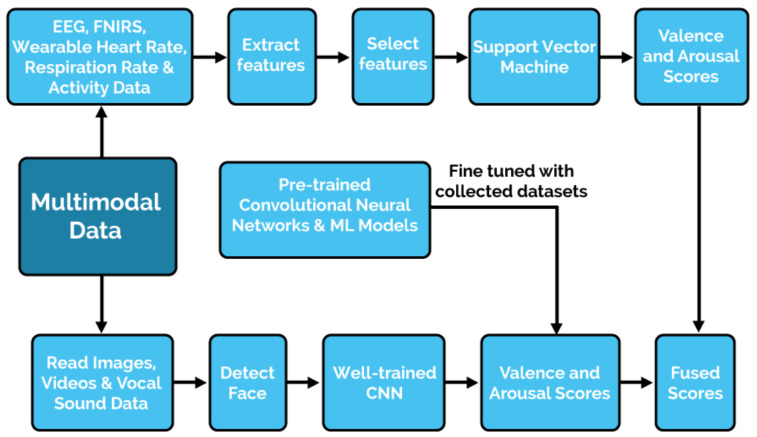
Multimodal affective state recognition data analysis workflow framework of the per-animal quantified approach. EEG—electroencephalogram; FNIRS—functional near-infrared spectroscopy; ML—machine learning; CNN—convolutional neural networks.

Establishing a distributed network of non-obtrusive, non-invasive sensors to collect real-time behavioural and physiological data from farm animals could be the initial step in the realization of framework development (Figure 2). Non-invasive sensors comprising video and thermal imaging cameras, microphones, and wearable TNO Holst 3-in-1 patches (monitoring heart rate, respiration rate, and activity) will help in the collection of data on behavioural and affective states. Data collected during natural behaviour, without any interference from experimenters, and the data collected during protocols in which defined positive and negative affective states will be induced in the animals using established protocols, including withholding high-value food from animals to induce disappointment [97]; placing animals in crowded situations to induce frustration [98,99]; and petting and socializing the animals to induce contentment [100,101] are some possibilities.

### 3.1. Classification and Annotation of Affective States and Behavioural Events

**Figure 3:**
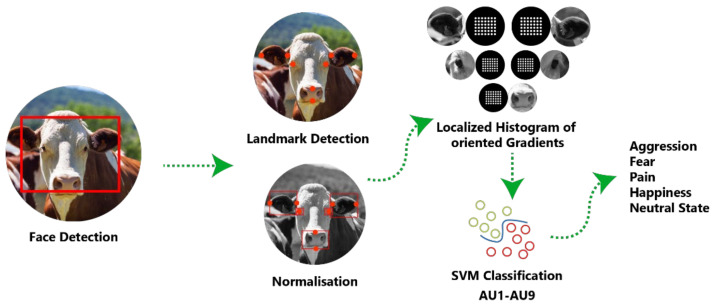
Pipeline of WUR Wolf (Wageningen University and Research—Wolf Mascot) automatic approach [102] in coding affective states from facial features of cows using machine learning models. SVM—support vector machine; AU—arbitrary units.

Common methods to identify affective and behavioural events in farm animals using sensors and AI enabled sensor data are: (a) An automatic affective state classification approach, capitalizing on preliminary work [102] conducted by FarmWorx of the Wageningen University. Pre-existing trained farm animals’ facial recognition platform such as WUR Wolf (Wageningen University and Research—Wolf Mascot) [102] can be used to classify changes in affective state over time in pigs and cows based on the video camera data (Figure 3). (b) Manual annotation of behavioural and emotional events in the data sets by ethologists and behavioural scientists with specific expertise in cow and pig behaviour, providing “gold-standard” annotated data sets. The annotators could potentially evaluate one category of behaviour (e.g., feeding, playing, resting) or affective state (e.g., fearful, happy, relaxed) at a time for all the animals under study, to maintain consistent scoring across animals. Krippendorff’s alpha coefficient could be calculated to compute the reliability across annotators and metrics, and to assess the influence of unequal sample sizes and disparities between dimensional and categorical variables on the results.

### 3.2. Sensor Network Fusion Protocols and Instrumentation Framework

Integrating heterogeneous sensor types into a multimodal network involves implementing a sensing platform capable of fusing data streams with differing precisions, update rates, and data formats to produce a common framework in which these data can be correlated and analysed. At present, there exist no platforms that possess the necessary functionality to correlate heterogeneous data streams, integrate diverse data sets, and identify data from individual animals [9].

There is a need for developing an instrumentation framework capable of integrating sensor data from diverse sensor types, opening the door to acquiring and analysing large data sets of multimodal sensor data on animal behaviour and affective state for the first time. It has to focus on establishing the hardware infrastructure to reliably gather large quantities of multimodal sensor data, along with the high-performance cloud server architecture to store and process these data.

In order to stream data in real time from multiple sensor types simultaneously, making use of long-range wide area network (LoRaWAN) communications technology, which is rapidly emerging as the state of the art in smart farming [103,104,105] would be ideal. LoRaWAN can wirelessly transmit data from 300 different types of sensors at a time, which will thereby allow the researchers to avoid the technical complexity and cost of a conventional wired setup. Extending the functionality of the LoRaWAN system to use low-energy Bluetooth technology, by increasing the length of time that data can be acquired from portable sensors before they need to be recharged [106,107,108] would save time and resource overload. To accelerate and facilitate the real-time analysis of the data, cloud servers connected via the internet must be used to store and process the data [33,109], avoiding the need to install complex and expensive computer servers at each individual farm site. The Microsoft AZURE platform is a commercial application that could allow seamless integration between the sensor data streams and the high-performance AI and ML methods used to analyse the data.

### 3.3. Build Predictive Models of Affective State and Behaviour

By using the data sets collected from the distributed sensor network, robust predictive models of farm animal behaviour and affective states can be built. Advanced statistical analyses applied to the annotated data set using supervised AI and ML methods, namely the Latent Growth Curve Modelling, Random Forest and Support Vector Machine models [110,111,112], offers established approaches in capturing and measuring patterns in dynamic interactive variables, such as behaviour and affective states of farm animals. These methods employed to extract features from the visual, thermal, auditory, physiological and activity sensor data, enables different behavioural and affective states to be distinguished with high accuracy, sensitivity, and selectivity [37,40,41,113].

Following the supervised training stage, unsupervised ML models applied aids in the determination of clusters of similar behavioural and affective state descriptors from unannotated sensor data obtained from farm animals [36,114,115]. These descriptors function as numerical “fingerprints” that allow distinct behavioural or affective states to be reliably identified, even in entirely novel data. The best features from each sensor modality corresponding to these descriptors can then be selected to define high-level specific indicators, which will then be fused to build an ML classifier-based model. There are two potential approaches to fusing sensor data from different modalities to predict behavioural and affective states which are (i) decision-level fusion, in which prediction scores from the unimodal models will be linearly combined; (ii) feature- and indicator-level fusion, in which feature vectors and indicators will be integrated across modalities to yield the prediction scores. The performance levels of different ML methods at estimating behavioural and affective states can be assessed using regression methods [116,117,118].

### 3.4. Challenges in the Quantification and Validation of Performance Models for Affective States Measurement

The assessment effectiveness of the platform at estimating behaviour and affective state in real time from farm animals is quite challenging. The predictive model can be evaluated by calculating its accuracy at estimating affective and behavioural states in novel data sets collected from the sensor network. In addition, the accuracy of the model can further be validated by correlating the affective and behavioural states it identifies against: (i) Quantitative assays of cortisol, lactate and oxytocin levels in blood and/or saliva samples from the animals [119,120]. These provide a reliable biochemical reference measure of emotional arousal and stress. (ii) Physiological indices associated with specific affective states in the animals, such as heart rate, respiratory rate, and body temperature. Physiological signals are more reflective of autonomic nervous system activity than non-physiological signals [121], such as facial expressions or vocalizations. Autonomic nervous system activation during emotional expression is involuntary in animals and therefore provides an unambiguous, quantitative reference measure for evaluating affective states.

#### 3.4.1. Sensor Durability

There is a risk that a wearable sensor cannot be attached securely to the animals, or the animals may damage the sensors by chewing or crushing. To mitigate the former, animal scientists or researchers could improve the adhesion protocol or use a belly belt, which is more secure.

#### 3.4.2. Low Sensitivity of the Model at Detecting Affective and Behavioural States

To address this, optimization of the AI algorithms and the sensors to increase sensitivity turn out to be useful.

#### 3.4.3. Lack of Correlation between Sensor Data and Biochemical Reference Values

Researchers collaborate with veterinarians to set up the biochemical validation assays.

#### 3.4.4. Limiting the Numbers of Animals Used in the Experiments

Increasing the sample size opens up ethical and practical issues [122]. The numbers of pigs and cows to be used in animal experiments should meet optimal research standards and experimental design but also meet the 3R (reduce, replace, refine) policies. Bayesian approaches could be used to increase the statistical power of the animal experiments using historical control data [122], while developing indices.

## 4. Scales and Indices of Animal Affective States—Bio-Instrumentation Perspective

**Figure 4:**
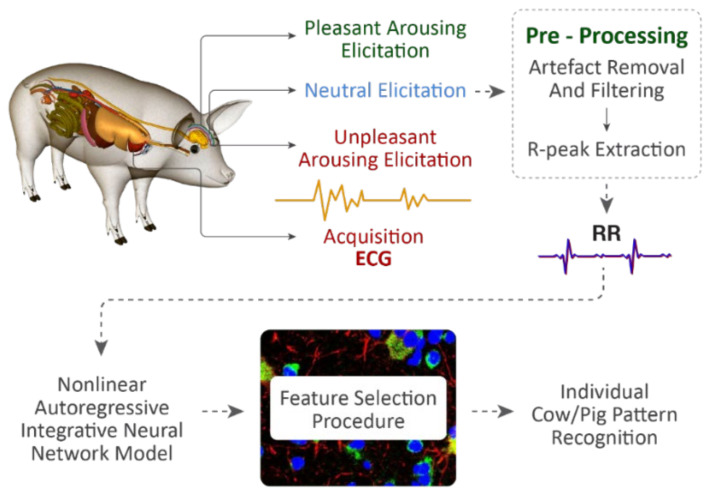
Overview of the farm animal affective measurement experimental set-up and block scheme of heart-rate signal processing and data classification chain.

The ability to accurately identify and measure affective states in farm animals is the gateway to creating more effective strategies for animal welfare. However, measuring affective states quantitatively remains a challenge, with no widely accepted methods or standards for doing so in farm animals [9]. While the recently developed Grimace Scales for pigs, cows and sheep provide a manual approach to quantifying pain via facial muscle movements [14,31,61], much work remains to develop comprehensive, quantitative measures for the full range of affective states. Standardized, scientifically validated scales and indices are of paramount importance to ensuring a consistently high quality of animal welfare across the livestock sector [1,5]. Using affective state paradigms previously applied to animals to collect behavioural and physiological data associated with positive and negative affective states (Figure 4) is the easy path towards development of scales or indices. Deep learning models can be trained to classify these affective states using the sensor collected data [45,92], and based on the insights gained, more accurate, scientifically validated scales and indices of animal welfare will be developed.

**Figure 5:**
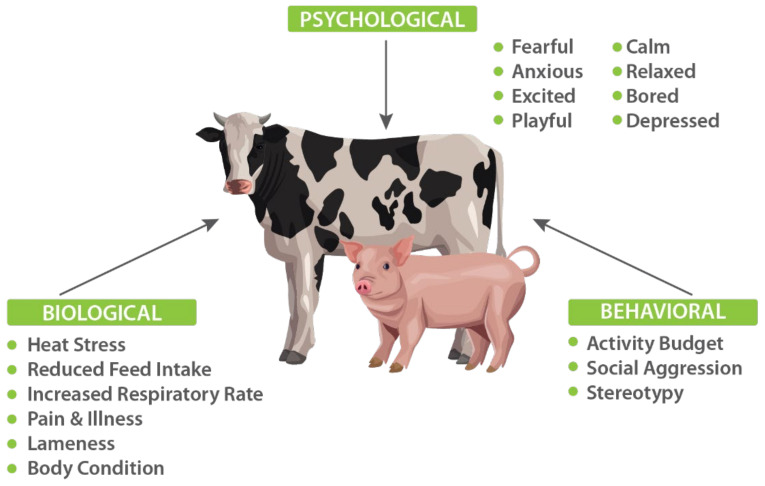
A framework for animal welfare assessment incorporating multimodal robust measures.

**Figure 6:**
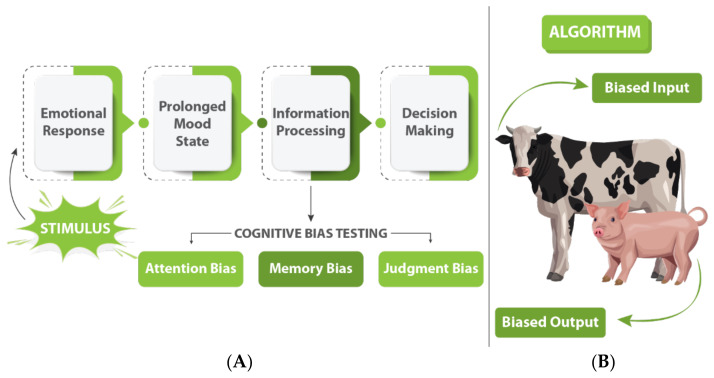
(**A**) Typical experimental paradigm for farm animal cognitive bias tasks. (**B**) Impact of bias in machine learning algorithm on animal emotion estimates.

By collecting data sets from a range of affective state paradigms to identify distinct biometric sensor signal signatures and then correlating with specific affective states in farm animals can provide a way to develop reliable indices [123]. Data Sets of multimodal sensor data can be collected and annotated with presumed affective states (Figure 5) based on the results of experimental paradigms that either directly induce affective states or assess them without inducing them. The following three paradigms can be shown as an example to directly induce affective states in farm animals:

(i) Video Stimulus Test: This test can measure approach/avoidance reactions in pigs or cows in response to videos of presumed positive and negative stimuli of varying valence and intensity [124,125,126]. Positive stimuli could involve unfamiliar pigs of the same breed, age and sex, while negative stimuli might involve a threat such as a barking dog [125]. The stimuli can then be projected onto a wall in a testing arena. The farm animals can then be tested either in pairs or alone to assess their levels of playfulness vs. nervousness during the trial [127].
(ii) Reward Gain/Loss Test: Anticipatory behaviour in relation to reward has been used to assess the affective state of cows [128] and pigs [129], with animals in a more negative state expected to show greater reward sensitivity and enhanced expression of anticipatory behaviours [96]. In addition to reward gain, reward loss can be used to induce behaviours related to a negative affective state by reducing or removing an expected reward in trained animals [128,129]. To conduct this test, cows and pigs would first be trained using a conditioning paradigm involving pairing a tone with access to the reward pen, where they will receive a food reward. Over the training period, the delay between the tone and reward will gradually be increased. Once the training is complete, anticipatory behaviours and associated biometric sensor data during the delay period can then be assessed. To induce reward loss, the size of the food reward will be reduced and the change in anticipatory behaviour over successive trials will be examined as the animals adjust their expectation of reward.
(iii) Social Recognition Test: Studies indicate that a number of animals, including sheep, are capable of recognising familiar faces of their conspecifics and human handlers [25,130]. Viewing familiar sheep but not goat faces was found to reduce behavioural and physiological indices of stress in sheep [25], thereby demonstrating a link between social recognition and emotional regulation. To conduct the test, the animals could be placed in a test pen for 15 min while an image of either a familiar face of a conspecific or a neutral stimulus (an inverted triangle of approximately the same contrast level) is projected onto a wall overlooking the test pen. Biometric sensor signals between the two conditions will then be compared, with the familiar face expected to induce a calmer state than the inverted triangle in the cows and pigs. To complement these tests, the current affective state of experimental animals can also be determined without deliberately inducing an affective state using the following paradigm:
(iv) Judgement Bias Task: This task can be used to evaluate the affective states of experimental cows and pigs (or other farm animals) by examining their response to an emotionally ambiguous stimulus [131]. Typically, the animal’s response is assessed (Figure 6) based on their response latency or a go/no-go decision, with a faster response or decision to approach indicating a positive affective state, and vice versa [132,133]. Animals will be pre-trained to respond differently to two distinct stimuli (e.g., low- and high pitch tones, or two spatial locations) and then tested using an ambiguous stimulus (e.g., an intermediate-pitch tone or intermediate location). Based on published studies in cows [134] and pigs [135], researchers and animal scientists are poised to explore both the auditory and spatial versions of the task to establish the optimal protocol: the former will test animals on a single feeding location coupled with a rewarded or unrewarded tone, while the latter will test them using two feeding locations, one rewarded and one unrewarded.

### Comparative Analysis of Annotated Affective and Behavioural Events in Data Sets

The annotation of behavioural and emotional events carried out by ethologists and behavioural scientists with specific expertise in cow and/or pig behaviour will provide “gold standard” data sets for the supervised training of AI and ML. The reliability of the annotation across annotators and metrics can be compared, and the influence of unequal sample sizes and disparities between dimensional and categorical variables on the results will provide reliable framework. The annotated biometric sensor data collected can be used to train a range of ML models, which will be iteratively improved to obtain a high accuracy, sensitivity and selectivity for the different affective states [37,40,41]. Three different types of ML model have previously been applied to accurately classify affective states from sensor data [136,137]: (i) Hybrid deep learning models, combining a convolutional neural network with a long short-term memory (LSTM) model to achieve multi-model data fusion; (ii) Multiple-fusion-layer based ensemble classifiers of stacked autoencoders; (iii) Combined extreme learning machine and support vector machine models. The transfer learning performance of the models [138] (their ability to generalise by classifying affective states from unfamiliar contexts) can be evaluated using a leave-one-out cross-validation procedure [139]. This typically involves training the models on a subset of data from all except one of the paradigms and then measuring the model’s performance on the “left-out” data [138].

Emotional contagion indicates the phenomenon of spontaneous spreading and automatic adoption of emotional state of another animal. Investigating the contributions of social interaction and emotional contagion at the herd level to affective states in individual farm animals [11,38,39,140,141,142,143,144] would be a requirement to strengthen the welfare monitoring framework reliability. Antagonistic social interactions, such as aggressive behaviours, are a serious health and welfare problem that affects not only the animals but also the animal caretakers. They include tail biting among pigs [145], feather pecking and cannibalistic behaviour among poultry [98], and microaggressions among cattle [146]. In contrast, synergistic interactions between individuals in a herd decrease stress, reduce inter-animal aggression, and help to prevent challenging or dangerous human–animal interactions from arising. Therefore, identifying the factors associated with antagonistic vs. synergistic interactions in a herd is an important step for developing effective and targeted animal welfare enhancement and intervention programs [147].

Development of social network analysis methods for analysing interactions within and outside the herd is an essential requirement in welfare monitoring platform development. To develop an analytical model building on the social network analysis (SNA) method to analyse quantitatively and objectively, the factors below should be considered.

(i) Inter-animal social interactions;
(ii) Emotional contagion (positive or negative) within the herd;
(iii) Human–animal interactions;
(iv) Non-social interactions (e.g., with feeding stations).

SNA focuses on analysing the structure of relationships between animals, using a graph theoretical approach in which agents (animal, human or site of interaction) are represented as nodes in a network, while types of interactions and their strengths are represented by edges. Interactions between agents will be determined by their spatial proximity based on the visual sensor data. When combined with the other multimodal sensor data and the annotations it will provide a rich picture of the physical, physiological, and behavioural events accompanying these interactions. This integration of multimodal sensor data into the SNA model developed here will allow in-depth analyses for the first time of how affective states influence and emerge from interactions, including the phenomenon of emotional contagion [11,141], and how interpersonal interactions (between specific individual animals) are maintained and develop over time.

The ‘Affective State’ models developed should pass the scientifically validated scales and indices of affective state and welfare. Currently, only physiological and behavioural measurements have been used to evaluate affective state and welfare in farm animals, and scientifically validated benchmarks are lacking [9]. Although Grimace Scales provide a well-defined and objective method for manually scoring facial muscle movements, they are limited to quantifying pain-related expressions [31]. Therefore, there is a pressing need to develop standards capable of measuring the full range of affective states in farm animals [17]. The accuracy of the models at classifying and quantifying different affective states can be efficiently validated using three reference standards:

(i) Grimace Scales for pigs and cows [14,31,61]
(ii) Standard blood biomarkers of stress or relaxation, including cortisol, lactate and oxytocin [25,78]
(iii) Physiological measures from wearable sensors (e.g., heart and respiratory rates), indexing autonomic nervous system activity [93]
(iv) Functional near-infrared spectroscopy (FNIRS) measurements, assessing cortical activity related to affective states [148].

The models would then be further refined based on comparisons to the references above, to develop scales and indices of affective state and welfare that are robustly cross validated. Finally, an animal welfare auditing platform is expected to be developed to make it straightforward for animal caretakers to visualize and act on the results from the scales and indices.

Some of the associated challenges are the subjectivity of affective and behavioural annotation by experts. This challenge can be mitigated through an interdisciplinary approach involving researchers across multiple backgrounds, to resolve any inconsistencies in the annotations made by experts vs. the trained models. However, the models and standards are essential for the future development of effective welfare monitoring platforms; thus, the risks justify the potential gain.

## 5. Digital Twin Systems to Report and Predict Affective States in Real Time

To respond rapidly to changes in the behaviour, affective state, and health of their animals, caretakers need to be continuously updated with the status of the animals under their charge. Digital twin models of individual animals promise a next generation approach to realizing this real-time flow of biometric information [47,48]. A digital twin is a “real-time” digital model of a physical entity that is updated continuously with data from the entity [149]. It simulates the inner dynamics and environmental interactions of the entity to identify patterns in its behaviour, learn cause–effect relationships, and suggest remedial actions to human operators, based on predictions. Although digital twin models have had a dramatic impact in the manufacturing, construction and healthcare sectors, by increasing efficiency and reducing costs, they are yet to be applied to the agricultural sector [48,150].

**Figure 7:**
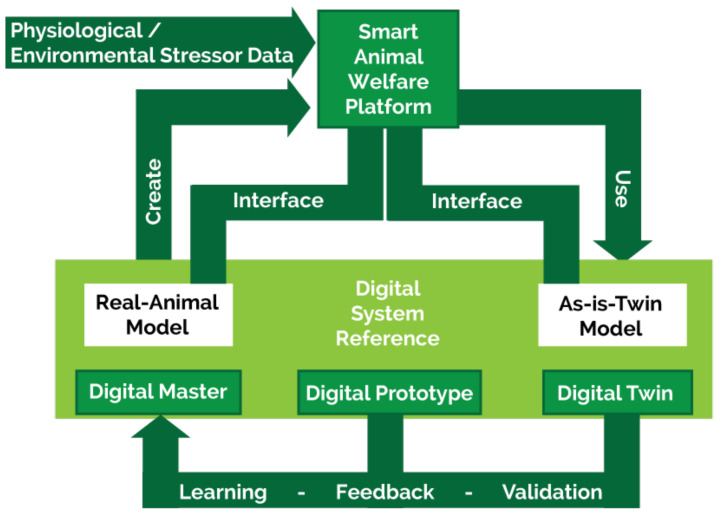
Digital Twin system reference architecture for smart animal welfare platform in predicting the behaviour of farm animals.

In prior theoretical work, I proposed an animal digital twin architecture composed of the following interlinked components (Figure 7) [48]:

(i) remote and wearable sensors that collect data from the animal;
(ii) cloud servers that interact with the sensors to receive, store, and process the sensor data, and to change sensor states;
(iii) AI models that learn to spot patterns in the data and ML models that make predictions;
(iv) a user interface, via which human caretakers receive and interact with the information and predictions of the digital twin.

By developing digital twins of animals, it will be possible to answer questions such as: What physical and social conditions best support the animal’s emotional wellbeing? How is this animal going to behave in the near future based on its prior behaviour? Digital twin models open novel data-driven approaches to modelling animal behaviour and affective state, which empower caretakers to provide individualized care to their animals based on continuous tracking and prediction of each animal’s behaviour and affective state. By displaying the information and predictions of the digital twin in an intuitive way on an interactive dashboard, caretakers will be better informed about their animals, enabling them to respond rapidly and effectively to mitigate distress and ensure their animals’ wellbeing.

**Figure 8:**
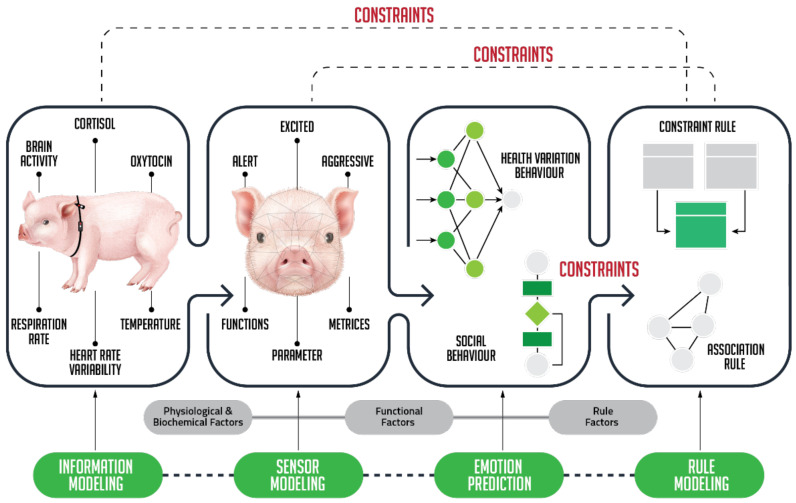
Sensor-based Digital Twin animal emotion modelling process.

Developing a digital twin architecture for modelling and predicting farm animal behaviour and affective state will build on the sensor and cloud computing infrastructure. For testing the capacity of the infrastructure to collect and process sensor data without interruption over periods of days, multimodal, biometric sensor data need to be continuously collected from a pilot group of farm animals. The sensor network will generally be comprised of video and thermal imaging cameras, microphones, and functional near-infrared spectroscopy (fNIRS) sensors, along with wearable skin impedance sensors and TNO Holst 3-in-1 patches (measuring heart and respiratory rates, and activity level) fitted to the animals. Radio-frequency identification (RFID) tags can also be used to reliably identify and locate individual animals [151]. Time-stamped sensor data captured from each of the animals and from videos will provide enriched information (Figure 8) to synchronize the digital twin representation with the animal itself.

The environmental context of the facility and its influence on the animal’s behaviour should be incorporated into the digital twin model. These environmental influences are important as they affect the animal’s behaviour: for example, cows tend to seek a secluded place during calving, while pigs may seek a warmer place in the pen because of airflow or ventilation issues. To account for this, environmental data must be continuously acquired from ventilation, light level, and feed and water intake sensors at the facility.

### 5.1. Development of Digital Twin Processing Pipeline for Classifying and Estimating Affective State and Behaviour in Real Time

Development of the sensor data processing pipeline (Figure 8) at the core of the digital twin model, involves pre-processing, modelling and simulation stages. Initially, temporal, and spatial data acquired from the animals could be pre-processed using moving-average and least-squares fitting algorithms, which have been applied successfully in prior work on affective state classification from biophysical signals [152]. To aggregate the large quantity of sensor data and to facilitate ML model training, a metric for classifying sensor data into positive, neutral, and negative affective states could be established based on the validated data sets and models.

The optimal approach to modelling affective state and behaviour within the digital twin using AI and ML models can be explored using deep neural network models [151], support vector machine (SVM), linear discriminant analysis (LDA), random forest (RF), and k-nearest neighbours (kNN). The best features selected from the AI models can be used to train ML models, such as the random forest regressor [112,153], which will simulate potential future behavioural and emotional scenarios. Using a basic output interface, human operators using the digital twin will be able to visualize and interact [154] with the predictions of the ML models. This will allow caretakers to act on the predictions made and to provide supervision to the digital twin to iteratively improve its performance.

The digital twin system can deepen our understanding of the factors contributing to physical and emotional resilience in the animals during their maturation. This in-depth understanding is important for making evidence-based changes to animal husbandry practices that can enhance animal welfare and facilitate the detection and prevention of disease [1,6]. To achieve this, the following indices can be compared between the experimental group of farm animals such as piglets and a control group without digital twins:

(i) Physical wellbeing, such as body weight and health/disease status;
(ii) Emotional wellbeing, such as heart rate variability and facial expression;
(iii) Social wellbeing, such as synergistic or antagonistic interactions with conspecifics;
(iv) Environmental factors, such as ventilation quality and light levels.

In addition, interventions made by the caretaker could be analysed to quantify if and how the digital twin supports caretaker decision-making to improve the welfare and the physical and emotional resilience of the animals. This ability will provide a robust pilot test of the potentials and challenges of using a digital twin to guide and improve animal caretaking decisions across a significant period of maturation in pigs.

To date there has been no attempt to develop a digital twin of a livestock animal. Therefore, the potential gain and the risk in the design and development of a farm animal digital twin are both high. Some risk assessments are given below.

#### 5.1.1. Interruption of the Continuous Data Feed

The digital twin system relies on a continuous data feed from sensors monitoring the physical entity. However, the wearable sensors attached to animals need to be changed every 7 days because of their limited battery life. This will make certain time lapses in the data collection, meaning that any data from this period will be lost. In addition to these battery issues, maintaining the continuous acquisition of data over the course of several weeks will be a significant technical challenge. This could potentially be overcome by data extrapolation methods and or advancements in the instrumentation approaches.

#### 5.1.2. Damage to Wearable Sensors

The wearable devices may be damaged or destroyed by the animals, especially by pigs who may chew or crush the devices during natural behaviours such as rooting. Therefore, adjustments should be made to make the wearable devices animal-friendly, so that it can withstand harsh environmental conditions such as mud/soil and allow the pigs to display their natural behaviours. To mitigate this challenge, the feasibility of a hard-packaged but skin compatible belly belt, which will enable the 3-in-1 sensor to be worn behind the pig’s right leg could be explored.

#### 5.1.3. Lack of Reference Models

This is a high-risk regarding development of digital twin of farm animals. There are currently no reference models available to directly guide or inform the research in the development of an animal twin. Initially, developing conceptual guidelines to support the implementation of digital twin models capable of measuring affective states, in addition to those for automated welfare monitoring systems could be established followed by actual development of a twin.

#### 5.1.4. Standardizing Outputs from the Digital Twin

It will be a challenge to standardize the measurement and prediction of animal behaviour and affective state, which is necessary to provide the animal caretakers with comparable decision-making information across different animal species and monitoring periods. Addressing this challenge would help to reduce biases in the caretaker’s view of animal welfare, reduce operating and maintenance costs, and improve farm management by intervening via organizational and technological means. To overcome this, the ISO framework established in the automation systems and integration as reference architecture (ISO/DIS 23247-2) could be deployed.

## 6. Conclusions

Because of the influence of insights into emotional intelligence in the decision making processes for autonomous monitoring of animal welfare, AI is becoming an inherent component of digital livestock farming. As a result, animal science relies on sensor technologies and data like never before. AI powered emotional measurement will significantly transform and influence how farmers and animal caretakers manage the emotions and interactions with farm animals. Enhanced understanding of farm animal emotional intelligence will assist farm managers to move towards the dual goal of increasing productivity and providing enriched quality of life experiences for animals. AI technologies offer a hands-on, realistic and practical approach to affective states recognition that assists animal caretakers and ethologists to comprehend the reasons why animals behave the way they do and how to maximize their welfare and productivity.

## Data Availability

Not applicable.
