# Correction: Neethirajan, S. Affective State Recognition in Livestock—Artificial Intelligence Approaches. *Animals* 2022, *12*, 759

**DOI:** 10.3390/ani12141856

**Published:** 2022-07-21

**Authors:** Suresh Neethirajan

**Affiliations:** Farmworx, Adaptation Physiology Group, Animal Sciences Department, Wageningen University and Research, 6700 AH Wageningen, The Netherlands; suresh.neethirajan@wur.nl

The authors wish to make the following correction to the original paper [1]:

## Figures Deletion

Figure 1 has been removed due to the fact that it is not relevant to the scope of the quantification of emotions measurement in farm animals. 

The authors apologize for any inconvenience caused and state that the scientific conclusions are unaffected. The original publication has been updated.

## Figures and Tables

**Figure 1:**
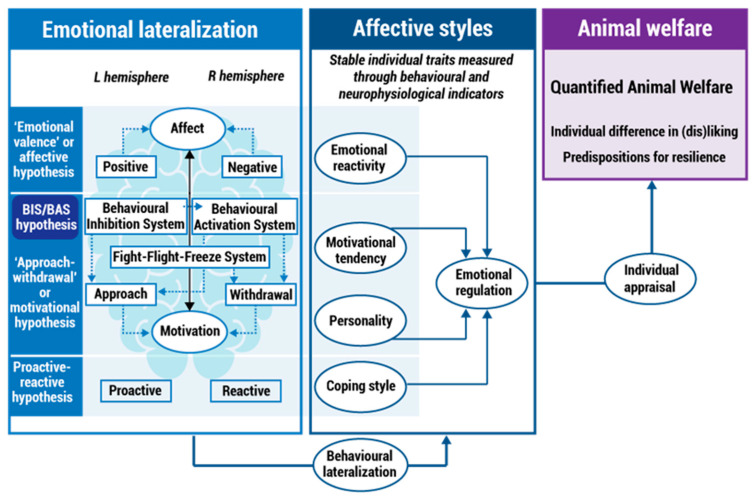
Framework for affective style measurement driven animal welfare research: Relationship between the farm animal emotional lateralization, animal welfare and the affective states. Lateralization: Asymmetrical representation of the control of emotions and processing in the animal brain. BIS—behavioural inhibition system; BAS—behavioural activation system. BIS—Behavioural Inhibition System; BAS—Behavioural Activation System. Reprinted from [44] Applied Animal Behaviour Science 237, 105279, Goursot, C.; Düpjan, S.; Puppe, B.; Leliveld, L. M. Affective styles and emotional lateralization: A promising framework for animal welfare research, 2021, Creative Commons Attribution (CC BY 4.0). https://doi.org/10.1016/j.applanim.2021.105279 (accessed on 2 October 2021). Text in the right hand box was changed from “Individualized welfare” to “Quantified Animal Welfare”.
